# Internal jugular vein versus subclavian vein as the percutaneous insertion site for totally implantable venous access devices: a meta-analysis of comparative studies

**DOI:** 10.1186/s12885-016-2791-2

**Published:** 2016-09-22

**Authors:** Shaoyong Wu, Jingxiu Huang, Zongming Jiang, Zhimei Huang, Handong Ouyang, Li Deng, Wenqian Lin, Jin Guo, Weian Zeng

**Affiliations:** 1 Department of Anesthesiology, State Key Laboratory of Oncology in South China, Collaborative Innovation Center for Cancer Medicine, Sun Yat-sen University Cancer Center, 651 Dongfeng East Road, Guangzhou, Guangdong 510060 People’s Republic of China; 2 Department of Anesthesiology, Shaoxing People’s Hospital (Shaoxing Hospital of Zhejiang University), Shaoxing, Zhejiang China; 3 Department of Minimal Invasive Intervention, Sun Yat-sen University Cancer Center, State Key Laboratory of Oncology in South China, Guangzhou, China; 4 Department of Anesthesiology, The First Affiliated Hospital of Soochow University, Suzhou, Jiangsu China

**Keywords:** Internal jugular vein, Subclavian vein, Totally implantable venous access device, Meta-analysis

## Abstract

**Background:**

A totally implantable venous access device (TIVAD) provides reliable, long-term vascular access and improves patients’ quality of life. The wide use of TIVADs is associated with important complications. A meta-analysis was undertaken to compare the internal jugular vein (IJV) with the subclavian vein (SCV) as the percutaneous access site for TIVAD to determine whether IJV has any advantages.

**Methods:**

All randomized controlled trials (RCTs) and cohort studies assessing the two access sites, IJV and SCV, were retrieved from PubMed, Web of Science, Embase, and OVID EMB Reviews from their inception to December 2015. Random-effects models were used in all analyses. The endpoints evaluated included TIVAD-related infections, catheter-related thrombotic complications, and major mechanical complications.

**Results:**

Twelve studies including 3905 patients published between 2008 and 2015, were included. Our meta-analysis showed that incidences of TIVAD-related infections (odds ratio [OR] 0.71, 95 % confidence interval [CI] 0.48–1.04, *P* = 0.081) and catheter-related thrombotic complications (OR 0.76, 95 % CI 0.38–1.51, *P* = 0.433) were not significantly different between the two groups. However, compared with SCV, IJV was associated with reduced risks of total major mechanical complications (OR 0.38, 95 % CI 0.24–0.61, *P* < 0.001). More specifically, catheter dislocation (OR 0.43, 95 % CI 0.22–0.84, *P* = 0.013) and malfunction (OR 0.42, 95 % CI 0.28–0.62, *P* < 0.001) were more prevalent in the SCV than in the IJV group; however, the risk of catheter fracture (OR 0.47, 95 % CI 0.21–1.05, *P* = 0.065) were not significantly different between the two groups. Sensitivity analyses using fixed-effects models showed a decreased risk of catheter fracture in the IJV group.

**Conclusion:**

The IJV seems to be a safer alternative to the SCV with lower risks of total major mechanical complications, catheter dislocation, and malfunction. However, a large-scale and well-designed RCT comparing the complications of each access site is warranted before the IJV site can be unequivocally recommended as a first choice for percutaneous implantation of a TIVAD.

## Background

Since Niederhuber et al. first introduced the totally implantable venous access device (TIVAD) at the MD Anderson Cancer Center in 1982 [1], TIVAD systems have gained worldwide popularity in oncology patients [2]. The number of implanted TIVADs is increasing, with more than 400,000 sold each year in the USA [3]. The use of a TIVAD allows for the long-term administration of venotoxic compounds, reduces the risk of infection, markedly alleviates the burden of intravenous therapy and thereby improves these patients’ quality of life, as this device does not require any external dressing. [3–5] Nevertheless, approximately 15 % of patients experience catheter-related complications [6]. The implantation of a TIVAD can be performed by different methods, such as percutaneous insertion and surgical venous cut-down [5, 7]. Even through, percutaneous TIVAD insertion has become the preferred method of implantation worldwide [5].

Several meta-analyses [8, 9] and the latest review [10] have recommended the routine utilization of ultrasound guidance in practice. With the help of ultrasound guidance, the percutaneous approach has the lowest rate of early complications [11]. Oncologists are most concerned with long-term complications occurring during the use of TIVADs [12]. Because the internal jugular vein (IJV) and subclavian vein (SCV) are the most common access sites to implant catheters in the superior vena cava (SVC) for long-term use [13, 14], it would be helpful to know which site is associated with fewer complications in the long-term follow-up.

Although several studies comparing the IJV and the SCV have been reported, most are small series of patients with conflicting results [15–18]. To date, neither valid recommendations nor guidelines concerning the choice of access site and long-term complications of TIVADs have been elaborated. In this meta-analysis, we sought to assemble the most robust dataset currently available to address a single focused clinical question: which access site, the IJV or the SCV, has fewer late complications for the percutaneous insertion of TIVADs?

## Methods

### Search strategy

We performed the meta-analysis in accordance with the Meta-analysis Of Observational Studies in Epidemiology (MOOSE) guidelines and the Preferred Reporting Items for Systematic Reviews and Meta-Analyses (PRISMA) statement [19, 20]. Eligible studies were searched in online databases including PubMed, Embase, Web of Science, and OVID EMB Reviews, from inception to December 2015. A variety of synonyms for “totally implantable venous device”, “internal jugular vein”, and “subclavian vein” were combined. The complete search process is presented in Table 1. A manual search of the citations and references in the articles retrieved for full review was conducted to identify the potentially eligible studies. No limitations were placed on the time period of the trial or the reporting language. Authors were contacted for additional information if necessary.

**Table 1:** Search process

Database	Search filter	Results
PubMed	("Catheterization, Central Venous/adverse effects"[Mesh] OR "Catheterization, Central Venous/methods"[Mesh] OR "Catheters, Indwelling/adverse effects"[Mesh]) AND ((totally implantable*[tiab]) OR (TIV*[tiab]) OR (port[tiab]) OR (ports[tiab])) AND ((jugular*[tiab]) OR (subclavian*[tiab]))	236 articles
Web of Science^a^	#1 TOPIC: (totally implantable venous port*) Timespan = All years Search language = Auto #2 TOPIC: (totally implantable venous device*) Timespan = All years Search language = Auto #3 TITLE: (port-a-cath* OR TIVA* OR port OR ports) Timespan = All years Search language = Auto #4 TOPIC: (jugul* OR subclavian*) Timespan = All years Search language = Auto #5 (#3 OR #2 OR #1) #6 (#5 AND #4)	865 articles
Embase	#1 implant* NEAR/5 (port OR ports OR device OR devices OR system OR systems) #2 TIVAP:ab OR TIVP:ab OR TIVAD:ab OR port:ab OR CVAP:ab #3 jugul*:ab OR subclavian*:ab #4 #1 OR #2 #5 #3 AND #4 AND ([article]/lim OR [article in press]/lim OR [conference abstract]/lim OR [conference paper]/lim OR [review]/lim) AND [humans]/lim	944 articles
All OVID Evidence-Based Medicine Reviews^b^	#1 (implant* and (port or device or system)).mp. [mp = ti, ot, ab, tx, kw, ct, sh, hw] #2 (TIVAP or TIVAD or TIVP or TICVP).mp. [mp = ti, ot, ab, tx, kw, ct, sh, hw] #3 (jugul* or subclavian*).mp. [mp = ti, ot, ab, tx, kw, ct, sh, hw] #4 #1 or #2 #5 #3 AND #4	61 articles

^a^Including Web of Science^TM^ Core Collection, BIOSIS preview®, Chinese Science Citation Database^SM^, Derwent Innovations Index^SM^, Inspect®, KCI-Korean Journal Database, MEDLINE®, SciELO Citation Index

^b^Including Cochrane DSR, ACP Journal Club, DARE, CCTR, CMR, HTA, and NHSEED

### Inclusion and exclusion criteria

All available randomized controlled trials (RCTs), non-randomized cohort studies that compared the IJV with the SCV as the puncture site for a TIVAD in all age groups, were included. Letters, editorials, case reports, review articles, and animal experimental studies were excluded. In order to make the clinical heterogeneity between studies smaller, the studies with follow-up less than 180 days were excluded. If a study investigated multiple access sites (IJV, SCV, and cephalic vein) [16, 18, 21], only the data from the IJV and the SCV were included.

### Data collection

Data extraction was performed by two independent authors (SYW and JXH). Agreement between the two reviewers was measured using the k statistic. Any discrepancies were resolved by discussion with the remaining authors. Demographics, clinical characteristics (age, brand of TIVAD used) and technique used (IJV percutaneous insertion, SCV percutaneous insertion, with or without ultrasound guidance or fluoroscopy) were collected. The complications of TIVAD were categorized into infectious complications, thrombotic complications, and mechanical complications [22, 23].

The primary outcomes were the incidence of TIVAD-related infections and thrombotic complications from the time of TIVAD insertion to TIVAD removal or the end of study; TIVAD-related infections were defined according to updated guidelines by the Infectious Diseases Society of America [24] and included pocket infection, local infection, and catheter-related bloodstream infection [25]. Catheter-related thrombotic complications were defined as a mural thrombus extending from the catheter into the lumen of a vessel and leading to partial or total catheter occlusion, with or without clinical symptoms (including fibrin sheath, deep vein thrombosis, major and complete thrombosis), [26, 27] which would be diagnosed using Doppler ultrasound, [15] follow-up chest radiography or chest computed tomography [17]. The secondary outcome was the rate of major mechanical complications after insertion of the TIVAD and follow-up. Major mechanical complications were defined in accordance with the Clavien-Dindo classification of surgical complications (grade III /IV/V) [28], including catheter malfunction (including infusion malfunction, aspiration malfunction, a combination of both, namely catheter occlusion [29]), catheter dislocation (also called malposition/migration; namely, the tip of catheter lying in a different vein from the intended superior vena cava [30]), catheter fracture (breakage or fracture of the catheter, including the breakage or disconnection of junction between the catheter and the reservoir, with or without embolism by catheter fragments), pinch-off syndrome, port rotation, port extrusion, hemorrhage, and extravasation. In addition, if there were more than three included studies and the complication was common, the data for a single major mechanical complication was pooled for meta-analysis. Late complications were unlikely to be due to the port implantation procedure itself [4], so immediate mechanical complications, such as pneumothorax, arterial puncture, and hematoma, which were procedure-related, were excluded in this meta-analysis. Other immediate mechanical complications such as primary malposition, which could be solved immediately with or without fluoroscopic control [15], were not included in this study. Above all, immediate mechanical complications were not included in this study. In addition, minor mechanical complications (Clavien-Dindo grade I/II), such as catheter looping, [31] were also excluded.

### Quality assessment

The quality of RCTs was assessed using the Cochrane Collaboration’s tool for assessing risk of bias guided by the Cochrane Handbook for Systematic Reviews of Interventions (version 5.1.0) [32]. Six domains were evaluated: sequence generation, allocation sequence concealment, blinding, incomplete outcome data, selective outcome reporting, and other sources of bias. The overall risk of bias in each study was assessed using the following judgments: low, moderate, or high, which was specified in the study by Ata-Ali [33].

The methodological quality of each nonrandomized observational study was evaluated by the Newcastle-Ottawa scale, which consists of three domains: patient selection (0–4 points), comparability of the study groups (0–2 points), and assessment of outcome (0–3 points) [34]. A quality score of 0–9 points was allocated to each nonrandomized study. RCTs with low risk of bias and nonrandomized studies achieving ≥ 7 points were considered to be of high quality.

### Statistical analysis

All of the available data were binary outcomes; therefore, they were combined as pooled odds ratio (OR) with 95 % confidence intervals (CIs). Heterogeneity of outcomes was diagnosed by *Q* statistics (with a significance level set at *P* = 0.10) and *I*^2^ statistics (>75 % indicating high heterogeneity) [35, 36]. The random-effects model was used in all analyses to produced more conservative and cautious estimates [9].

Subgroup analyses were conducted for the outcomes of TIVAD-related infections, catheter-related thrombotic complications, and total major mechanical complications. Data stratified according to patient’ age, whether antibiotic prophylaxis was used, whether ultrasound guidance was used, were analyzed to investigate clinical factors affecting our outcomes. Sensitivity analyses were conducted to examine the robustness of the effect by alternatively using a fixed-effects model. We also did sensitivity analyses according to two different study designs (RCT and non-randomized cohort study). Only outcomes with more than one studies were included in the sensitivity analyses. Publication bias was assessed using Egger regression asymmetry test [37]. A two-tailed *P* value < 0.05 was considered statistically significant, except otherwise specified. Statistical analysis was performed using R software (https://www.r-project.org; last access 29 March 2016) and Stata software version 12 (StataCorp LP, College Station, USA).

## Results

A total of 2106 potentially eligible studies were initially identified, and 2078 were excluded after screening the titles and abstracts. The remaining 28 articles were fully reviewed. Of these, 14 were excluded because the data were not extractable, two studies were duplicate reports with different outcomes, and one was a RCT with only 30 days follow-up which did not fulfill the criteria of minimum follow-up of 180 days in this meta-analysis. In addition, one study [23] was identified from the citations of the study by Araujo [15].

**Fig. 1:**
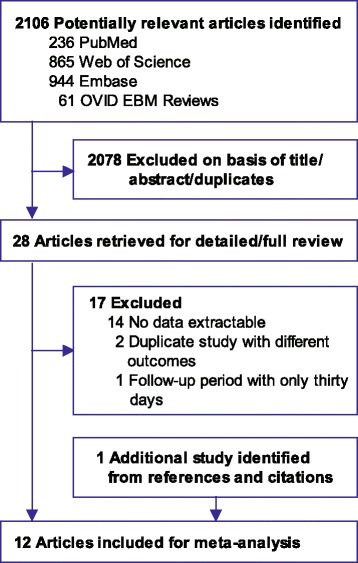
Flowchart of the literature search and selection process

Therefore, 12 studies [15–18, 21, 23, 31, 38–42] including 3905 patients (1824 patients in the IJV group and 2081 patients in the SCV group) published from 2008 to 2015 were included (Fig. 1). Agreement on study selection between the two reviewers was high (k = 0.94). Among the included studies, there were three RCTs [16, 39, 41] and two prospective non-randomized controlled trials [15, 31]. The remaining seven studies [17, 18, 21, 23, 38, 40, 42] were retrospective. The characteristics of the included studies are summarized in Table 2.

**Table 2:** Baseline characteristics of studies included in the meta-analysis

Study	Country	Design	Participants					Use of heparin flushing	Antibiotic prophylaxis	Ultrasound guidance	Matching criteria^a^	Follow-up^b^, IJV/SCV
			Age, yr (median/mean)	Range	TIVAD	IJV	SCV					
Araujo^c^ [15], 2008	Portugal	PC	55.5 (median)	15–83	Mini-sitimplant	512	551	Y	N	N	1,2,3,4,5	244/363d (median)
Biffi [16], 2009	Italy	RCT	51.9 (mean)	18–75	Bard Port	117	123	Y	NR	Only for SCV	1,2,4,6,7,8	384/360d (median)
Plumhans [31], 2011	Germany	PC	56 (mean)	18–85	Bard Port	44	94	Y	NR	Only for IJV	7,8	6 mo (mean)
Aribaş [38], 2012	Turkey	RC	53.8 (mean)	16–84	Polysite	248	99	Y	NR	Y	1,2,4,7,8	219.5d (mean)
Ribeiro [39], 2012	Brazil	RCT	< 18 yr	NR	NR	34	43	Y	Y	N	1,2,4,6,7,8	14.8/12.6 mo (mean)
Vetter [21], 2013	Germany	RC	53 (mean)	2–84	INTRAPORT	71	32	Y	Y	N	1,2	451d (mean)
Liu^d^ [40], 2014	China	RC	45.4 (mean)	8–86	Bardport	222	398	Y	NR	N	1,2,3,4	1079.3/995.2d (mean)
Miao [41], 2014	China	RCT	58.1 (mean)	25–81	NR	107	107	Y	Y	Only for IJV	1,2,3,4,8	215/209d (mean)
Nagasawa^e^ [17], 2014	Japan	RC	64 (median)	25–85	BARD X-port isp	136	97	NR	NR	Only for IJV	3	566/402d (mean)
Ozbudak [42], 2014	Turkey	RC	56.38 (mean)	14–83	FB Medical/Districlass medical SA	178	224	Y	N	Y for some patients	3,8	507d (median)
Wu [18], 2014	Taiwan	RC	57.7 (mean)	0.5–94	Arrow/Bard/ Tyco	63	234	Y	NR	N	NA	4.5 yr (mean)
Jung [23], 2015	Korea	RC	59 (median)	1–82	Bard Port	92	79	NR	NR	N	1,2,4,7	278d (median)

*Abbreviations*: *d* days, *mo* months, *N* No, *NR* data not reported, *PC* prospective cohort study, *RC* retrospective cohort study, *US* ultrasound guidance, *Y* Yes, *yr* years

^a^for matching criteria: 1 = age; 2 = gender; 3 = completion of the TIVAD insertion; 4 = site of primary malignancy; 5 = time of surgery; 6 = side; 7 = TIVAD outer diameter; 8 = coagulation parameters; 9 = body mass index

^b^Mean or median dwell time

^c^Only 512 and 551 patients were included in the analysis for group IJV and SCV respectively

^d^One catheter fracture due to iatrogenic injury was not included in the analysis

^e^One case of pin hole leakage in the IJV arm was included in the major mechanical complications

The risk of bias of the RCTs included in this meta-analysis is summarized in Table 3. Of the three RCTs, two [16, 39] were considered to have low risk of bias, and one [41] had a moderate risk of bias. Among the nine nonrandomized studies, seven [15, 17, 18, 21, 23, 40, 42] were considered to be of high quality, and two [31, 38] were regarded as being of low quality (Table 4).

**Table 3:** Cochrane summary assessment of risk of bias for included RCTs

Study	Sequence generation	Allocation concealment	Blinding	Incomplete outcome data	Selective outcome reporting	Other sources of bias	Risk of bias^a^
Biffi, 2009	yes	yes	yes	yes	yes	no	low
Ribeiro, 2012	yes	uncertain	yes	yes	yes	yes	low
Miao, 2014	uncertain	uncertain	yes	yes	yes	no	moderate

^a^Five or six domains with “yes” represents low risk of bias; three or four domains with “yes” represents moderate risk of bias; two or fewer domains with “yes” represents high risk of bias

**Table 4:** Newcastle-Ottawa Scale for nonrandomized cohort studies

Study	Selection				Comparability	Outcome			Quality score
	Representativeness of the Exposed Cohort	Selection of the Non-Exposed Cohort	Ascertainment of Exposure	Demonstration That Outcome of Interest Was Not Present at Start of Study	Comparability of Cohorts on the Basis of the Design or Analysis	Assessment of Outcome	Was Follow-Up Long Enough for Outcomes to Occur	Adequacy of Follow Up of Cohorts	
Araujo, 2008	1	1	1	1	1	1	1	0	7
Plumhans, 2011	1	1	1	1	1	1	0	0	6
Aribaş, 2012	1	1	1	1	1	1	0	0	6
Vetter, 2013	1	1	1	1	1	1	1	1	8
Liu, 2014	1	1	1	1	0	1	1	1	7
Nagasawa, 2014	1	1	1	1	0	1	1	1	7
Ozbudak, 2014	1	1	1	1	1	1	1	1	8
Wu, 2014	1	1	1	1	1	1	1	1	8
Jung, 2015	1	1	1	1	2	1	1	1	9

### Primary outcomes

**Fig. 2:**
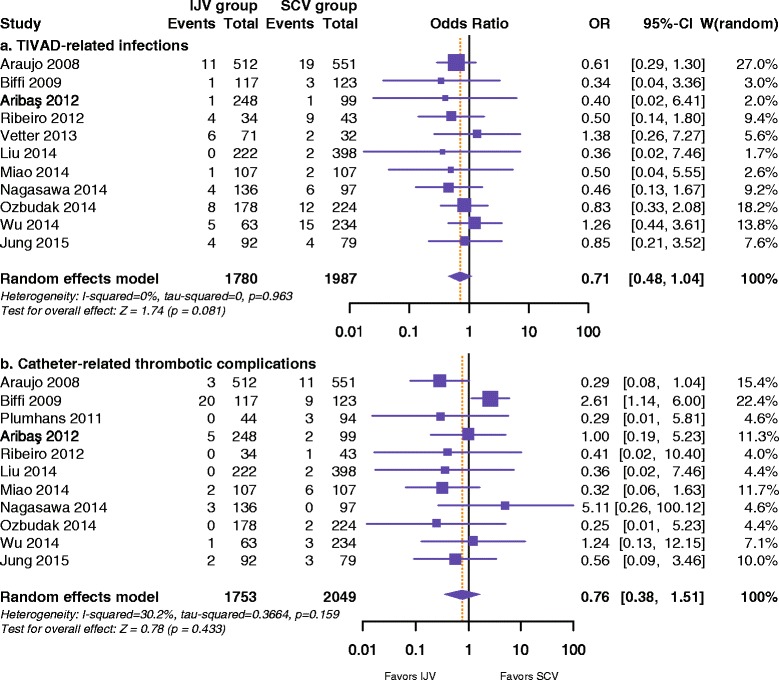
Forest plot and meta-analysis of TIVAD-related infections (**a**) and catheter-related thrombotic complications (**b**)

The pooled data from 11 studies [15–18, 21, 23, 38–42] that assessed TIVAD-related infections (Fig. 2a) in 3767 patients showed no significant differences between the IJV and SCV groups (2.53 % and 3.77 %; OR 0.71, 95 % CI 0.48–1.04; *P* = 0.081) with no significant between-study heterogeneity (*I*^2^ = 0.0 %; *P* = 0.963). Catheter-related thrombotic complications were reported in 11 studies [15–18, 23, 31, 38–42] that investigated 3802 patients (Fig. 2b). There were no significant differences between the IJV and SCV groups (2.05 % and 2.05 %; OR 0.76, 95 % CI 0.38–1.51; *P* = 0.433), with no significant between-study heterogeneity (*I*^2^ = 30.2 %; *P* = 0.159).

### Secondary outcomes

**Fig. 3:**
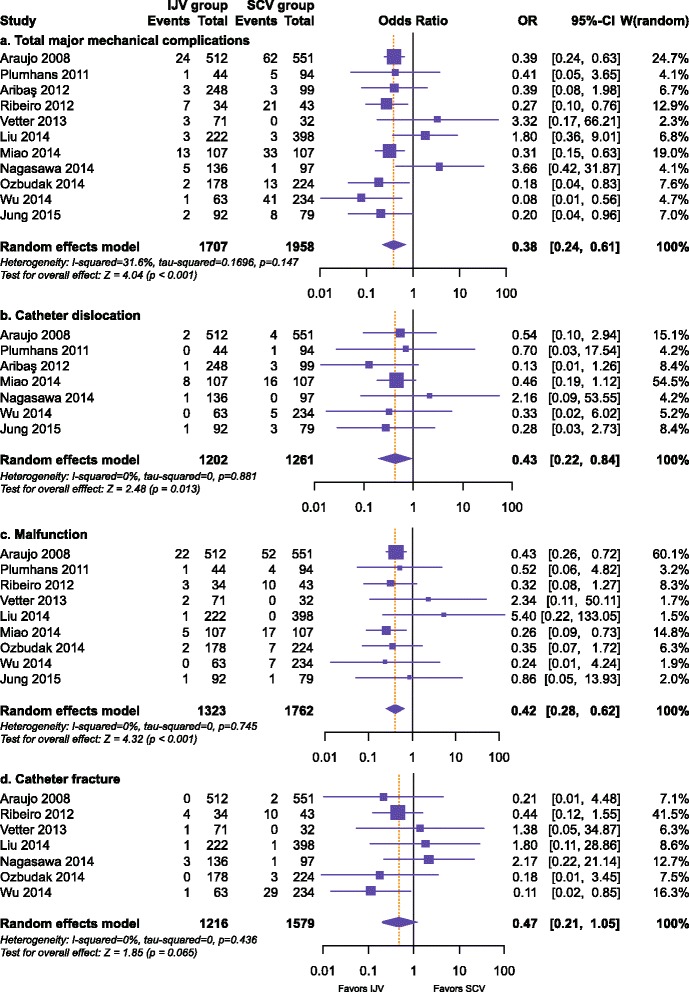
Forest plot and meta-analysis of major mechanical complications, including total major mechanical complications (**a**), catheter dislocation (**b**), malfunction (**c**), and catheter fracture (**d**)

Data on major mechanical complications were available in 11 studies, [15, 17, 18, 21, 23, 31, 38–42] which evaluated 3665 patients (Fig. 3a). The rate of total major mechanical complications was significantly higher in the SCV group than in the IJV group (3.75 % in the IJV group and 9.70 % in the SCV group; OR 0.38, 95 % CI 0.24–0.61; *P* < 0.001), with low between-study heterogeneity (*I*^2^ = 31.6 %; *P* = 0.147). Additionally, there were three major mechanical complications that more than three studies reported: catheter dislocation, malfunction, and catheter fracture. In other words, these three complications were common. As a result, the data for the three major mechanical complications were pooled for meta-analysis.

Seven studies [15, 17, 18, 23, 31, 38, 41] that reported on catheter dislocation in 2463 patients showed a significant difference favoring the IJV group (1.08 % in the IJV group and 2.54 % in the SCV group; OR 0.43, 95 % CI 0.22–0.84; *P* = 0.013) (Fig. 3b). Nine studies [15, 18, 21, 23, 31, 39–42] that assessed 3085 patients reported on malfunction, and the difference was statistically significant in favor of the IJV (2.80 % in the IJV group and 5.56 % in the SCV group; OR 0.42, 95 % CI 0.28–0.62; *P* < 0.001) (Fig. 3c). Pooling the data of seven studies [15, 17, 18, 21, 39, 40, 42] including 2795 patients that reported on catheter fracture showed no significant difference between the two groups (0.82 % in the IJV group and 2.91 % in the SCV group; OR 0.47, 95 % CI 0.21–1.05; *P* = 0.065) (Fig. 3d). All of the three major mechanical complications showed no significant heterogeneity (Fig. 3b, c, and d).

### Subgroup analyses

Subgroup analyses showed that use of antibiotic prophylaxis did not influence the incidence of TIVAD-related infections (Table 5). In the subgroup analyses of ultrasound guidance, only one study [38] used ultrasound to guide the TIVAD insertion for all patients, and six studies [15, 18, 21, 23, 39, 40] used anatomical landmark technique for all patients (Table 1). The results showed that the use of ultrasound guidance did not affect the risks of TIVAD-related infections and catheter-related thrombotic complications; however, it moderated the effect size of total major mechanical complications (Table 5). In addition, subgroup analyses stratified by the patients’ age showed no change in our conclusions for the outcomes of TIVAD-related infections and catheter-related thrombotic complications; however, in the subgroup of adults, the risk of total major mechanical complications was not significantly different between the two groups with higher heterogeneity (*I*^2^ = 56.5 %; *P* = 0.100) (Table 5), indicating that heterogeneity in the total major mechanical complications was due to other factors, rather than patients’ age.

**Table 5:** Subgroup analyses comparing IJV versus SCV^a^

Group	TIVAD-related infections				Catheter-related thrombotic complications				Total major mechanical complications			
	N	OR (95 % CI)	*I* ^2^(%)	*P*_heterogeneity_	N	OR (95 % CI)	*I* ^2^(%)	*P*_heterogeneity_	N	OR (95 % CI)	*I* ^2^(%)	*P*_heterogeneity_
Overall	11	0.71 (0.48–1.04)	0.0	0.963	11	0.76 (0.38–1.51)	30.2	0.159	11	0.38 (0.24–0.61)	31.6	0.147
Use of antibiotic prophylaxis												
Yes	3	0.69 (0.27–1.76)	0.0	0.611	NA	NA	NA	NA	NA	NA	NA	NA
No	2	0.69 (0.39–1.24)	0.0	0.618	NA	NA	NA	NA	NA	NA	NA	NA
Use of ultrasound guidance												
Yes	1	0.40 (0.02–6.41)	NA	NA	1	1.00 (0.19–5.23)	NA	NA	1	0.39 (0.08–1.98)	NA	NA
No	6	0.76 (0.47–1.24)	0.0	0.798	5	0.44 (0.18–1.05)	0.0	0.860	6	0.38 (0.18–0.79)	46.3	0.098
Age Group												
< 18 yr	1	0.50 (0.14–1.80)	NA	NA	1	0.41 (0.02–10.40)	NA	NA	1	0.27 (0.10–0.76)	NA	NA
≥ 18 yr	3	0.44 (0.16–1.22)	0.0	0.972	4	1.13 (0.28–4.61)	56.8	0.073	3	0.61 (0.15–2.56)	56.5	0.100

*Abbreviations*: *N* Number of studies, *NA* not applicable, *yr* years old

^a^All these analyses were performed with random-effects model

### Sensitivity analyses

Sensitivity analysis by alternatively using a fixed-effects model did not show any relevant influence on all of the outcomes except catheter fracture, which showed a reduced risk in the IJV group (OR 0.38, 95 % CI 0.18–0.78; *P* =0.008) with low heterogeneity (*I*^2^ = 0.0 %; *P* = 0.436) (Table 6). In the sensitivity analyses, RCTs and non-randomized studies showed the same results for the overall OR estimates for TIVAD-related infections, catheter-related thrombotic complications, total major mechanical complications, and malfunction (Table 6).

**Table 6:** Sensitivity analyses comparing IJV versus SCV

Outcomes	OR (95 % CI)			
	Base case^a^	Using fixed-effects model	RCTs included^a^	Non-randomized cohort studies included^a^
TIVAD-related infections	0.71 (0.48–1.04)	0.70 (0.47–1.03)	0.47 (0.17–1.28)	0.76 (0.50–1.16)
Catheter-related thrombotic complications	0.76 (0.38–1.51)	0.91 (0.57–1.43)	0.90 (0.17–4.68)	0.56 (0.27–1.16)
Total major mechanical complications	0.38 (0.24–0.61)	0.36 (0.26–0.49)	0.30 (0.17–0.53)	0.44 (0.22–0.88)
Catheter dislocation	0.43 (0.22–0.84)	0.43 (0.23–0.83)	NA^b^	0.40 (0.15–1.07)
Malfunction	0.42 (0.28–0.62)	0.42 (0.28–0.62)	0.28 (0.12–0.64)	0.47 (0.30–0.74)
Catheter fracture	0.47 (0.21–1.05)	0.38 (0.18–0.78)	NA^b^	0.50 (0.15–1.61)

*Abbreviation*: *NA* not applicable

^a^Random-effects model was used in these analyses

^b^Sensitivity analysis was not conducted because only one study was included

### Publication bias

**Fig. 4:**
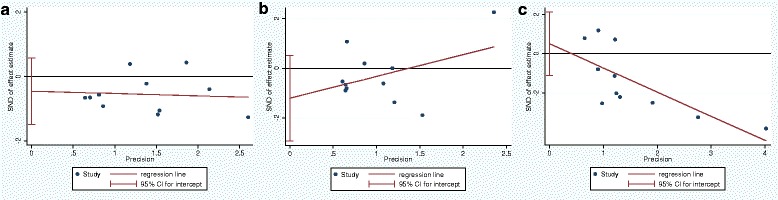
Egger funnel plots for TIVAD-related infections (**a**), catheter-related thrombotic complications (**b**), and total major mechanical complications (**c**)

Publication bias was assessed by Egger regression asymmetry test, which did not suggest any significant publication bias for TIVAD-related infections (*P* = 0.343), catheter-related thrombotic complications (*P* = 0.147), total major mechanical complications (*P* = 0.502), catheter dislocation (*P* = 0.959), malfunction (*P* = 0.265), and catheter fracture (*P* = 0.730) among the included studies. Egger funnel plots for TIVAD-related infections, catheter-related thrombotic complications, and total major mechanical complications were shown in Fig. 4.

## Discussion

This meta-analysis of three RCTs and nine non-randomized cohort studies, all of which included a total of 3905 patients, compared the efficacy of the IJV and the SCV as the percutaneous access site for a TIVAD. The results suggested that compared with the SCV, the IJV seems to be a safer venous access site with significantly reduced major mechanical complications. To be more specific, the IJV is associated with a lower risk of catheter dislocation and malfunction. We found no significant differences in TIVAD-related infections and thrombotic complications. On subgroup analyses, the use of antibiotic prophylaxis did not influence the incidence of infectious complications; the use of ultrasound guidance did not affect the risks of TIVAD-related infections and catheter-related thrombotic complications, but it moderated the effect size of total major mechanical complications. On sensitivity analyses, the overall estimates of all endpoints except catheter fracture remain robust by alternatively using a fixed-effects model; both RCTs and non-randomized cohort studies showed the same results for TIVAD-related infections, catheter-related thrombotic complications, and total major mechanical complications.

Easy and reliable vascular access is particularly important in cancer patients. The introduction of TIVADs has made the treatment of oncology patients more comfortable and convenient, because dressing is not required and daily activities of the arms need not be restricted once the port is implanted [43]. Compared with external indwelling catheters, advantages of the TIVAD include reduced risk of infection, greater patient acceptance and requiring less maintenance [3, 44]. However, like other short-term central venous catheters, TIVAD also presents risks itself after long-term indwelling.

The rate of TIVAD-related infections in our study was 2.53 % in the IJV group and 3.77 % in the SCV group, which was consistent with the reported results (3–10 %) of a recent review [3]. Subgroup analysis showed that the use of antibiotic prophylaxis did not influence the overall estimates for infections. We did not find a significant difference between the two groups in terms of TIVAD-related infections. Because patients with cancer are susceptible to infections due to immune depression and neutropenia [3, 13], we also suggest that measures should be taken to reduce the risk of infections, including sterile precautions during TIVAD insertion, and optimal aseptic catheter maintenance [12, 45, 46].

The incidences of catheter-related thrombosis in this meta-analysis were both 2.05 % in the IJV and in the SCV group, which were consistent with the results (0.3–28.3 %) of a review by Verso [47]. Thrombosis represents a major problem in oncology practice [48]. Cancer patients are usually at increased risk of venous thrombosis [49]. Although anticoagulant prophylaxis is controversial, routine heparin flushing of the port seems to be sufficient to prevent thrombosis formation [12]. In this meta-analysis, the majority of included studies reported on use of heparinized saline flushing regularly for primary prevention of catheter-associated thrombosis, and only two studies [17, 23] did not mention the use of heparin for routine maintenance of the TIVAD, which, however, did not mean heparin was not used. Actually, prophylactic heparin flushing has become the routine of clinical practice [50]. Consequently, subgroup analysis stratified by whether heparin was used was not conducted. Furthermore, placement of the catheter tip low in the SVC or at the atriocaval junction resulted in a lower risk of thrombosis than placement higher in the SVC [51, 52]. As a result, the use of fluoroscopy after implantation was recommended to identify tip position and ensure adequate catheter length (catheter tip below the T3 level) [52]. When thrombosus occurs, we may resort to medical treatment (anticoagulant agents or thrombolytic drugs) or even remove the TIVAD [48].

Catheter dislocation (also defined as a secondary malposition) can occur months after implantation of the TIVAD if the catheter tip is dislocated from its original position [2, 13]. Radiological control of the catheter tip using chest fluoroscopy after implantation is mandatory [12, 53]. In fact, all the included studies in this meta-analysis used fluoroscopy to confirm the catheter tip in the right place. The reason why catheter dislocation is more common in SCV group is still unclear. However, according to a retrospective study by Paleczny [14], spontaneous dislocation of the vascular port and catheter tip associated with changes in body position was found by chest radiograph in two patients with the catheters placed only in the SCV rather than in the IJV group. This phenomenon indicates that TIVAD insertion via the SCV route may be more subject to spontaneous dislocation when changing body position in daily life.

The pinch-off syndrome is specifically associated with the SCV approach [54]. Due to the compression of an implantable port between the clavicle and the first rib, the pinch-off syndrome can result in mechanical compression and shearing forces on the catheter lines [55], which may lead to malfunction, damage, and even fracture of the catheter after material fatigue [56], with embolization in the lung vascular bed. Pinch-off syndrome serves as a warning prior to catheter fracture, a rare but serious complication [57]. We confirmed that compared with IJV, SCV was associated with more incidences of major mechanical complications and many (malfunction, damage and catheter fracture) may be due to pinch-off syndrome.

Our meta-analysis is unique and presents important implications for clinicians in that, to our knowledge, it is the first study to systematically summarize the association of venous access sites for percutaneous implantation of a TIVAD and long-term morbidity. We used a comprehensive search strategy and systematic review method, following the MOOSE guidelines and the PRISMA statement. We limited heterogeneity by including only studies with more than 180 days follow-up. Furthermore, we redefined the outcome of malfunction to cover all aspects of catheter malfunctioning, namely infusion and aspiration malfunction as well as a combination of both [29], thereby avoiding potential heterogeneity in the endpoint of malfunction. Moreover, heterogeneity was low to moderate in the analyses of all outcomes, suggesting that variations in findings are compatible with chance alone and not likely to be caused by genuine differences between studies [58].

Our study has the following limitations. First, the majority of included studies were not RCTs and often presented a small sample size. They were carried out in hospitals with different protocols and likely different levels of physician expertise. Second, the definitions of endpoints such as TIVAD-related infections, catheter-related thrombotic complications, were not clearly described in some studies; however, studies were pooled irrespective of their definitions of these endpoints. The heterogeneity in endpoint reporting of the primary studies should be considered as a limitation. Third, in the subgroup analysis, ultrasound guidance diminished the advantage of IJV for the outcome of total major mechanical complications. However, this result should be interpreted with caution, because only one study was included in the subgroup of ultrasound guidance. Fourth, the implantation of a TIVAD can be performed by surgical venous cut-down technique and percutaneous approaches [43], and the results of our meta-analysis only apply to percutaneous approaches. Fifth, some definitions of mechanical complications (port rotation, port extrusion, hemorrhage, and extravasation) were not sufficiently described; these outcomes were included in the outcome of total major mechanical complications and were not individually pooled for meta-analysis. Sixth, because the raw data of the included studies were not available and both arms were comparable in terms of the follow-up period in each study, the results of the analysis did not take into account of the number of catheter days. However, as the cumulative risk of infectious, thrombotic, and mechanical complications increased with increasing catheter exposure, the complications might have been underestimated due to the relatively short follow-up period in some studies [31, 41].

## Conclusions

In conclusion, in the present meta-analysis comparing the IJV and the SCV as a venous access site for percutaneous insertion of a TIVAD, we identified a better choice of the IJV in terms of the incidence of major mechanical complications (catheter dislocation and malfunction), but we did not find any statistically significant differences in TIVAD-related infections and thrombotic complications. Given the inherent limitations of the included studies, the findings from our study must be confirmed and updated in a large-scale and well-designed RCT with long-term follow-up.

## Abbreviations

CI: Confidence interval
IJV: Internal jugular vein
OR: Odds ratio
RCT: Randomized controlled trial
SCV: Subclavian vein
SVC: Superior vena cava
TIVAD: Totally implantable venous access device
